# Effects of mild calorie restriction on anxiety and hypothalamic–pituitary–adrenal axis responses to stress in the male rat

**DOI:** 10.1002/phy2.265

**Published:** 2014-03-20

**Authors:** Rachel Kenny, Tara Dinan, Guohui Cai, Sarah J. Spencer

**Affiliations:** ^1^ School of Health Sciences and Health Innovations Research Institute (HIRi) RMIT University Melbourne Vic. Australia

**Keywords:** Anxiety, calorie restriction, hypothalamic–pituitary–adrenal (HPA) axis

## Abstract

Chronic calorie restriction (CR) is one of the few interventions to improve longevity and quality of life in a variety of species. It also reduces behavioral indices of anxiety and influences some stress hormones under basal conditions. However, it is not known how CR influences hypothalamic–pituitary–adrenal (HPA) axis function or if those on a CR diet have heightened HPA axis responses to stress. We hypothesized elevated basal glucocorticoid levels induced by CR would lead to exacerbated HPA axis responses to the psychological stress, restraint, in the male rat. We first confirmed rats fed 75% of their normal calorie intake for 3 weeks were less anxious than ad libitum‐fed (AD) rats in the elevated plus maze test for anxiety. The anxiolytic effect was mild, with only grooming significantly attenuated in the open field and no measured behavior affected in the light/dark box. Despite elevated basal glucocorticoids, CR rats had very similar hormonal and central responses to 15‐min restraint to the AD rats. Both CR and AD rats responded to restraint stress with a robust increase in glucocorticoids that was resolved by 60 min. Both groups also showed robust neuronal activation in the paraventricular nucleus of the hypothalamus and in other stress‐ and feeding‐sensitive brain regions that was not substantially affected by calorie intake. Our findings thus demonstrate chronic mild CR is subtly anxiolytic and is not likely to affect HPA axis responses to psychological stress. These findings support research suggesting a beneficial effect of mild CR.

## Introduction

Calorie restriction (CR) is one of the few interventions that can actually prolong life and it does so in a multitude of species (McCay et al. 1989; Fontana et al. 2010). CR has also been associated with a large number of positive indices of quality of life. It attenuates weight gain, improves insulin sensitivity and beta‐cell dysfunction in type 2 diabetics (Jackness et al. 2013), protects against cancer, and prevents cardiovascular disease (Weiss and Fontana 2011; Pallavi et al. 2012). Calorie restriction is also known to reduce indices of anxiety in some behavioral tests in animal models (Levay et al. 2007; Guccione et al. 2012; Willette et al. 2012). Despite these known positive effects, CR is extraordinarily difficult to maintain (Marinilli Pinto et al. 2008). Humans consistently relapse when voluntarily undertaking a CR diet (Marinilli Pinto et al. 2008) and rodents, primates, flies, and other species will maintain their calorie intake higher than the optimal for promoting longevity and health if given ad libitum access to food (Fontana et al. 2010). In light of these observations, we hypothesized CR imposes a significant stressor to the animal that encourages it to feed again despite the benefits.

There are some data to indicate CR may alter some stress‐related pathways. For instance, it is clearly established that CR can enhance basal corticosterone concentrations (Chacon et al. 2005; Levay et al. 2010; Tomiyama et al. 2010). Levay and colleagues have shown a dose–response relationship between CR and corticosterone in rats such that the greater the CR is, the higher the basal circulating corticosterone concentration (Levay et al. 2010). However, the effects of CR on hypothalamic–pituitary–adrenal (HPA) axis function are by no means clear. Adrenocorticotropic hormone (ACTH), the “upstream” hormone responsible for stimulating glucocorticoid release after stress, is either reduced (Chacon et al. 2005) or unchanged (Levay et al. 2010) following CR, whereas corticotropin‐releasing hormone (CRH) levels in the hypothalamus are attenuated, suggesting HPA axis responses could be as well (Brady et al. 1990; Lindblom et al. 2005; Levay et al. 2010). Human studies report no increase in perceived stress with CR alone (Ho et al. 2007; Tomiyama et al. 2010). Furthermore, studies of behaviors related to HPA axis function, such as anxiety, usually report a profile consistent with reduced stress reactivity (Landgraf and Wigger 2002). Thus, at least under basal (unstressed) conditions, rats with mild (25 or 50%) CR are less anxious in an open field test, spending more time in the center of the arena (Levay et al. 2007).

It remains, therefore, unclear if the CR‐induced increase in corticosterone is symptomatic of heightened stress, or represents enhanced, protective, negative feedback mechanisms that ultimately dampen stress responsiveness. In this study, we aimed to investigate the effects of mild CR on anxiety and basal HPA axis function in the same study. We also aimed to examine the effects of CR on the HPA axis under stress conditions.

## Methods

### Animals

We conducted all procedures in accordance with the National Health and Medical Research Council Australia Code of Practice for the Care of Experimental Animals. All procedures were approved by the RMIT University Animal Ethics Committee.

We obtained 72 adult male Wistar rats 327–445 g from the Animal Resources Centre, WA, Australia. On arrival at the RMIT University Animal Facility, we pair‐housed the rats according to weight, the heaviest being paired with the next heaviest and so forth. We then housed the rats at 22°C on a 12 h light/dark cycle (0700–1900 h). We gave all the rats 2 weeks to acclimatize to their new environment, during which time they were all given ad libitum access to standard pelleted rat chow (Meat Free Rat and Mouse Diet, Specialty Feeds, WA, Australia; Table 1) and water. During week two of the acclimatization phase, the rats, and their food, were weighed daily to establish basal food intake.

**Table 1:** Calculated composition of the diet (Meat Free Rat and Mouse Diet, Specialty Feeds, WA, Australia)

	Unit	Composition
Calculated nutritional parameters		
Protein	%	20
Fat	%	4.8
Crude fiber	%	4.8
Acid detergent fiber	%	7.6
Neutral detergent fiber	%	16.4
Total carbohydrate	%	59.4
Digestible energy	MJ/kg	14
Total calculated energy from protein	%	23
Total calculated energy from lipids	%	12
Calculated total minerals		
Potassium	%	0.82
Calcium	%	0.8
Phosphorous	%	0.7
Sulfur	%	0.2
Magnesium	%	0.2
Sodium	%	0.18
Iron	mg/kg	200
Manganese	mg/kg	104
Zinc	mg/kg	90
Copper	mg/kg	23
Molybdenum	mg/kg	1.2
Cobalt	mg/kg	0.7
Iodine	mg/kg	0.5
Selenium	mg/kg	0.4
Cadmium	mg/kg	0.05
Calculated total vitamins		
Vitamin A	IU/kg	10950
Vitamin D	IU/kg	2000
Choline	mg/kg	1640
Niacin	mg/kg	145
Vitamin E	mg/kg	110
Vitamin B1	mg/kg	80
Pantothenic acid	mg/kg	60
Vitamin B2	mg/kg	30
Vitamin B6	mg/kg	28
Vitamin K	mg/kg	20
Folic acid	mg/kg	5
Biotin	*μ*g/kg	410
Vitamin B12	*μ*g/kg	150

### Calorie restriction

After the 2 weeks acclimatization period, we retained 50% of the rats on their ad libitum chow diet (ad libitum‐fed; AD) and gave the other 50% of the rats a diet that was restricted to 75% of their usual food intake (calorie restricted; CR) based on number of grams consumed in week two of the acclimatization period. We weighed the rats and gave them food at 0900 h daily, that is, 2 h after lights on. This feeding regime was chosen to avoid the rats being food‐deprived during subsequent behavioral and stress tests. Although all food was gone in the CR group by the following day, we did note there was food remaining in the cage just prior to lights out in every case, indicating food was still available during their normal feeding time. We kept the rats in pairs for these experiments to avoid the stress effects associated with single‐housing. A 3‐week calorie restriction was chosen to allow us to compare our findings with previously published results [e.g., (Levay et al. 2007)].

### Open field test for anxiety and locomotor activity

On day 18 of their AD or CR diets, we tested a subset of the rats (*n* = 18 AD and 18 CR) in the open field test for anxiety and locomotor activity as described previously (Spencer et al. 2005; Spencer and Tilbrook 2009). The open field was made of wood, painted black, and was 60 × 60 cm with 50‐cm high walls. We filmed each test and later scored it for locomotion (distance traveled), number of entries into the middle of the arena, vertical exploration (rearing), and frequency of grooming bouts, in a period of 10 min.

### Elevated plus maze test for anxiety

On day 21 of their AD or CR diets, that is, 3 days after the open field test, we tested these rats for 5 min in the elevated plus maze test for activity and anxiety in a novel environment, as described previously (Spencer et al. 2005; Spencer and Tilbrook 2009; Bulfin et al. 2011). The plus maze was of gray plastic and was raised 50 cm above the floor. It consisted of two opposite open arms of 50 × 15 cm and two closed arms of the same dimensions with 15‐cm high walls. We filmed each test and later scored each rat for the number of entries into and percentage time spent in each of the open and closed arms. These rats were also used for corticosterone measurements.

### Light/dark box test for anxiety

To examine performance in an additional test of anxiety, we tested a separate cohort of rats (*n* = 10 AD and 18 CR), at day 21 of their AD and CR diet, in the light/dark box test for anxiety (Onaivi and Martin 1989). We placed each rat in an enclosed (dark) arena (43.5 × 21.5 × 30 cm) and filmed and later scored it for the latency to move into, and the time spent exploring the high‐light arena (45 × 23.5 × 30 cm) in a 10 min trial. These rats were also used for immunohistochemistry and corticosterone measurements.

All behavioral tests were scored, by an experimenter blinded to the treatment groups, using Ethovision software (Ethovision XT; Noldus Information Technology; Wageningen, The Netherlands). The arenas were thoroughly cleaned with 70% ethanol between trials.

### HPA axis responses to stress

On day 22 of their AD and CR diets, we examined HPA axis responses to stress in these rats. We first extracted a baseline blood sample from each rat via tail nick. We quickly took each rat from its cage and nicked the end of the tail with a sharp razor blade to extract a ~20 *μ*L baseline blood sample into a heparinized capillary tube. Each sample was collected within 3 min of nicking the tail to minimize any handling effects on the corticosterone levels detected in the sample (Vahl et al. 2005). The pairs of rats were then allocated randomly into “nonstressed” and “stressed” groups. We gave each “stressed” rat a 15 min restraint stress [restraint of the rat in a ventilated Perspex tube, 7 cm in diameter, 24 cm in length, with an adjustable restraining length between 10 and 18 cm (Crane et al. 2005; Spencer and Tilbrook 2009)], and took blood samples at 30, 60, and 120 min after the onset of the restraint (*n* = 15 AD and 18 CR stressed). The baseline sample was collected immediately prior to stress onset. We took similar blood samples from the nonstressed rats, but this group was not given restraint (*n* = 9 AD and 16 CR nonstressed per group). Blood samples were kept on ice until the end of the experiment, when they were centrifuged and the plasma aliquots stored at −20°C until assayed.

At 120 min after the onset of the restraint, or equivalent in nonstressed animals, and immediately after the final blood sample, we deeply anesthetized the rats with Lethabarb (~150 mg/kg pentobarbitone sodium, intraperitoneal). We removed the adrenal glands for weighing and perfused the rats transcardially with phosphate‐buffered saline (PBS; 4°C, pH 7.4) followed by 4% paraformaldehyde in PBS (4°C, pH 7.4). We then removed the brains and postfixed them for 4 h in the same fixative before placing them in cryoprotectant with 20% sucrose in PBS (4°C). We subsequently cut forebrains into 40‐*μ*m coronal sections using a cryostat. All experiments were initiated between 0900 and 1200 h to limit potential effects of circadian rhythms on any parameters measured.

### Assessment of neuronal activation after stress

We assessed neuronal activation on the basis of positive Fos‐immunoreactivity, seen as a black deposit in the nucleus as previously described (Spencer et al. 2004a,b; Mouihate et al. 2010) (*n* = 8 AD and nine CR nonstressed, *n* = 9 AD and eight CR stressed). A one‐in‐four series of forebrain sections from each animal was incubated in primary Fos antibody (O/N; 1:10,000; rabbit polyclonal; Santa Cruz Biotechnology, Santa Cruz, CA), then in secondary antibody (1.5 h; 1:500; biotinylated anti‐rabbit; Vector Laboratories, Burlingame, CA) and in an avidin‐biotin horseradish peroxidase (HRP) complex (ABC; 45 min; Vector Elite kit; Vector). The sections were then incubated in nickel diaminobenzidine (DAB) to visualize the HRP activity, seen as a black nuclear deposit. The reactions were terminated once an optimal contrast between specific cellular and nonspecific background labeling was reached. Sections from each treatment group were processed simultaneously. Sections were mounted on polylysine‐coated slides, dehydrated in a series of alcohols, cleared in Xylene, and coverslipped.

### Corticosterone assay

We used a standard corticosterone enzyme immunoassay kit (Abnova Corp., Taipei, Taiwan) to assess plasma corticosterone. The interassay variability for this assay was 7.2% coefficient of variation (CV), intra‐assay variability 4.8% CV, and lower limit of detection 40 pg/mL. Samples from all treatment groups were assayed together in duplicate.

### Data analysis

An experimenter, blinded to the group treatments, carried out counts of cells positive for Fos‐immunoreactivity in the medial parvocellular (mp), magnocellular (mg), and dorsal parvocellular (dp) paraventricular nucleus of the hypothalamus (PVN) over two sections, in the arcuate nucleus (ARC) over two sections, in the paraventricular nucleus of the thalamus (PVT) over six sections, in the medial (MeA) and central (CeA) nuclei of the amygdala over four sections, and in the dorsal (d) and ventral (v) bed nucleus of the stria terminalis over four sections 160 *μ*m apart. Regions were defined according to the Paxinos and Watson rat brain atlas (Johnson et al. 2012). Counts were summed to derive a sampled total for each region. Photomicrographs were taken using an Olympus BX41 microscope (Melbourne, Vic., Australia) and DP72 digital camera with CellSens image capture software v1.6 (Olympus, Melbourne, Vic., Australia), and were cropped and adjusted for intensity and contrast in Corel Draw X6 (Ottawa, ON, Canada). Any intensity and contrast enhancements were performed identically on all photomicrographs.

We compared AD and CR body weight change from baseline (day 1 of their allocated diet) using an analysis of variance (ANOVA) with repeated measures, with diet as the between factor and time (day) as the repeated measure. When a significant interaction was found between diet and time, we performed Student's unpaired *t*‐tests for each time point. We compared each parameter of the elevated plus maze, open field, light/dark box, and counts of Fos‐immunoreactive cells between groups using two‐way ANOVAs with diet and stress as between factors. Where significant interactions were found, we then performed Tukey post hoc tests. We compared corticosterone concentrations between the groups using two‐way ANOVAs with repeated measures with diet and stress as between factors and time (min) as the repeated measure. We also compared the areas under the curve for the corticosterone response using two‐way ANOVAs with diet and stress as between factors and followed this with Tukey post hoc tests. Data are presented as the mean ± SEM. Statistical significance was assumed when *P *<* *0.05.

## Results

### Calorie restriction

**Figure 1:**
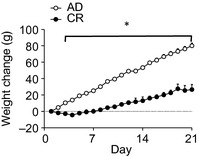
Weight gain from day 1 of experiment in ad libitum‐fed rats (AD) and rats given 75% of their normal food intake Chronic calorie restriction (CR). Weights are significantly different between the groups from experimental day 2. Data are mean ± SEM. *P <* *0.05.

The 25% calorie restricted diet caused a significant attenuation in weight gain compared with the AD group that was evident as early as the second day (Fig. 1. *F*_21,714_ = 116.66, *P *<* *0.001). Starting weights between the groups were not different for each cohort (cohort 1: AD 357 ± 3.9 g, CR 363 ± 4.8 g; cohort 2: AD 414.4 ± 4.6 g, CR 412.7 ± 3.5 g).

### Elevated plus maze test for anxiety

**Figure 2:**
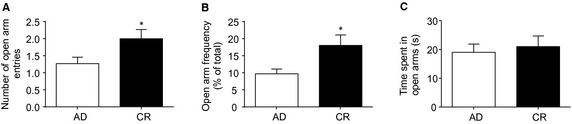
Elevated plus maze test for activity and anxiety. Chronic calorie restriction (CR) rats entered the open arms more often (A) and more frequently (B) than AD. However, the time spent in the open arms was not different between the groups (C). Data are mean ± SEM. *P <* *0.05.

To investigate the effects of a mild calorie restricted diet on anxiety behaviors, we tested our rats in the elevated plus maze test for activity and anxiety. There was no difference between the groups in total arm entries (AD: 12.7 ± 1.0; CR: 11.1 ± 1.1). We found CR rats entered the anxiety‐provoking open arms of the plus maze more often than AD (*t*_34_ = 2.05, *P *=* *0.048, Fig 2A). They also entered the open arms more frequently as a function of their total arm exploration, indicative that they were less anxious in the test (*t*_34_ = 2.44, *P *=* *0.02, Fig 2B). However, there were no significant differences between the groups in the time spent in the open arms (Fig. 2C).

### Open field test for anxiety and locomotor activity

**Figure 3:**
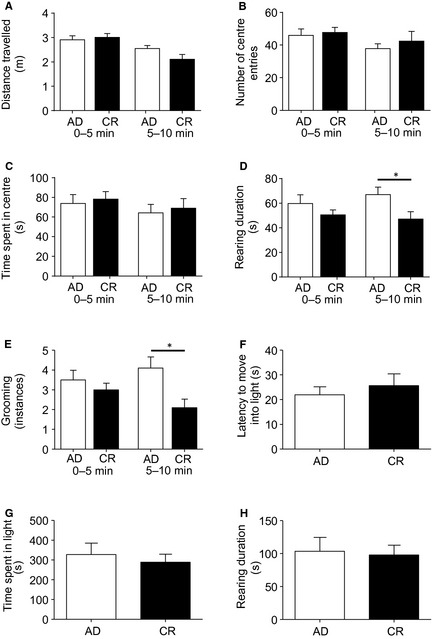
Open field and light/dark box tests for activity and anxiety. Chronic calorie restriction (CR) and AD rats displayed similar locomotory activity (A), center entries (B), and time spent in the center (C) of the open field. Chronic calorie restriction rats performed less vertical exploration (D) and groomed less (E) than the AD. Chronic calorie restriction (CR) and AD rats also had similar latencies to enter the light arena (F), time spent in the light (G), and vertical exploration in the light (H) in the light/dark box. Data are mean ± SEM. *P <* *0.05.

We also investigated anxiety behavior in the open field test for anxiety and locomotor activity. The CR rats tended to move around the open field less than the AD in the second 5 min of their test, but this was not statistically significantly different (*t*_34_ = 1.86, *P *=* *0.07, Fig 3A). There were no differences in the number of entries into the anxiety‐provoking center of the arena, or the time spent in the center (Fig. 3B, C). The CR rats spent significantly less time in vertical exploration than AD in the second 5 min of the test, indicative of enhanced anxiety (*t*_34_ = 2.33, *P *=* *0.026, Fig 3D). However, the CR also groomed themselves less often in the second 5 min of the test, a measure indicative of reduced anxiety (*t*_34_ = 2.33, *P *=* *0.008, Fig. 3E).

### Light/dark box test for anxiety

To further investigate the effects of mild calorie restriction on anxiety behaviors, we tested our rats in the light/dark box test for anxiety. In this test, there were no differences between the two groups of rats in their latency to move into the high‐light arena, time spent in the light, vertical exploration in the light (Fig. 3F–H), or instances of grooming (not shown).

### HPA axis responses to stress

**Figure 4:**
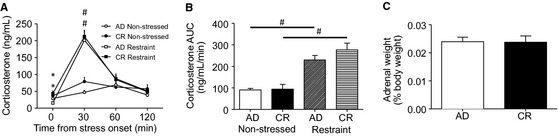
Plasma corticosterone responses to restraint. Plasma corticosterone levels were higher under basal conditions in the Chronic calorie restriction (CR) than the AD, but the groups had a similar absolute (A) and area under the curve (B) increase in corticosterone in response to stress. Chronic calorie restriction and AD rats also had similar adrenal weights as a percentage of their total body weight (C). Data are mean ± SEM. *P *<* *0.05. *significantly different from AD. ^#^significantly different from nonstressed.

The CR diet significantly elevated corticosterone levels under basal conditions (*F*_3,54_ = 6.7, *P *=* *0.012). However, there were no differences in the magnitude of the corticosterone response to stress or the timing of the resolution of the response. Both groups had a significant increase in corticosterone at 30 min after stress onset (*F*_3,42_ = 28.60, *P *<* *0.001) and this had returned toward baseline by 60 min (Fig. 4A, B). A significant effect of stress, but not of diet, was also seen with the area under the curve analysis (*F*_3,42_ = 23.88, *P *<* *0.001). There were also no differences in adrenal weights as a percentage of the total body weight between the groups (Fig. 4C).

**Figure 5:**
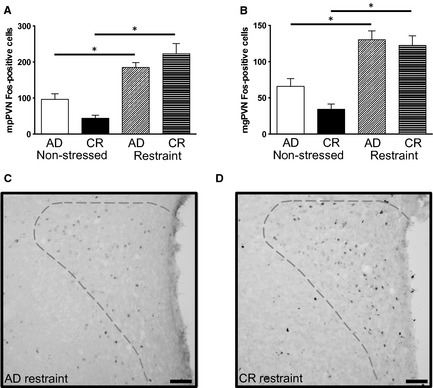
Central neuronal activation in response to restraint. Restraint stress induced similar neuronal activation in the medial parvocellular (mp) paraventricular nucleus of the hypothalamus (PVN) (A) and magnocellular (mg) PVN (B) PVN in AD and Chronic calorie restriction (CR) rats. C, D) Photomicrographs of the PVN from representative AD (C) and CR (D) restraint‐treated rats. Dashed lines show regions counted; dorsal parvocellular = top, mg = left, mp = right. Data are mean ± SEM. *P <* *0.05. Scale = 100 *μ*m.

The CR and AD rats also had similar levels of neuronal activation in relevant stress‐sensitive brain regions after restraint stress. Both groups showed the expected neuronal activation in response to restraint in the mpPVN (*F*_3,40_ = 60.40, *P *<* *0.001; Fig. 5, Table 2), the apex of the HPA axis, and there was no significant difference between the CR and AD groups with post hoc tests either under basal conditions or after stress. Restraint stress also activated the mg and dp regions of the PVN and did so similarly in all groups. There was a noteworthy trend towards fewer activated mpPVN neurons under basal conditions in the CR rats, but this was not statistically significant with post hoc tests. There was also something of a trend towards more neurons activated in the mpPVN after stress in the CR. Although the groups were again not significantly different with post hoc tests, there was a significant interaction between diet and stress in this region (*F*_3,30_ = 6.94, *P *=* *0.013) reflecting a larger change in neuronal activation after restraint stress in the CR rats than in the AD.

**Table 2:** Neuronal activation in response to restraint stress in brain regions associated with stress and feeding in AD and Chronic calorie restriction (CR) rats

Brain region	AD nonstressed	CR nonstressed	AD stressed	CR stressed	Main effect
mpPVN	96.4 ± 15.4	43.9 ± 8.5	185 ± 13.6^a^	223.4 ± 28.0^a^	Stress: *F*_3,30_ = 60.403, *P *<* *0.001; *Diet: NSD Diet and* Stress: *F*_3,30_ = 6.94, *P *=* *0.013
mgPVN	65.9 ± 10.8	34.2 ± 7.2	130.6 ± 11.9^a^	122.3 ± 13.5^a^	Stress: *F*_3,30_ = 48.20, *P *<* *0.001 Diet: NSD Interaction: NSD
dpPVN	22.6 ± 4.7	10.4 ± 3.1	25.1 ± 3.5	22.0 ± 4.5	Stress: NSD Diet: *F*_3,30_ = 3.75, *P *=* *0.062 NSD Interaction: NSD
MeA	423.8 ± 74.5	274.9 ± 55.6	797.7 ± 48.2^a^	982.1 ± 144.9^a^	Stress: *F*_3,30_ = 31.86, *P *<* *0.001 Diet: NSD Interaction: NSD
CeA	9.8 ± 2.0	27.3 ± 8.7	55.0 ± 11.2^a^	75.0 ± 17.5^a^	Stress: *F*_3,30 _= 16.39, *P *<* *0.001 Diet: NSD Interaction: NSD
dBNST	11.4 ± 1.8	21.1 ± 3.3	41.2 ± 4.9^a^	59.1 ± 8.7^a^	Stress: *F*_3,30 _= 36.67, *P *<* *0.001; Diet: *F*_3,30_ = 6.14, *P *=* *0.02 Interaction: NSD
vBNST	136.8 ± 22.9	119.4 ± 21.3	385.2 ± 91.6^a^	330.5 ± 65.7	Stress: *F*_3,30_ = 16.47, *P *<* *0.001 Diet: NSD Interaction: NSD
ARC	182.6 ± 33	78.2 ± 10.2	319.1 ± 43.1	327.1 ± 59.0^a^	Stress: *F*_3,30_ = 23.75, *P *<* *0.001 Diet: NSD Interaction: NSD
PVT	171.6 ± 27.6	92.8 ± 23.5	226.3 ± 31.7	295.6 ± 42.6^a^	Stress: *F*_3,30_ = 16.45, *P *<* *0.001 Diet: NSD Interaction: NSD

NSD, not significantly different; N = eight AD and nine CR nonstressed, *n* = 9 AD and eight CR stressed.

a Significantly different from nonstressed of the same diet.

Other regions of the brain associated with the response to psychological stress, the MeA, CeA, d, and vBNST were also not different between the CR and AD groups in terms of their neuronal activation after restraint. MeA, CeA, and d and vBNST all responded to restraint with an increase in neuronal activation and did so to a similar degree in the AD and CR groups (Table 2). In the dBNST, we saw a significant effect of both stress and diet (Stress: *F*_3,30_ = 36.67, *P *<* *0.001; Diet: *F*_3,30_ = 6.14, *P *=* *0.02), with a tendency towards more neurons activated under basal and poststress conditions in the CR, but there were no significant differences with the post hoc tests.

We also examined neuronal activation under basal and restraint‐stimulated conditions in the ARC nucleus, because of its role in feeding (Cowley et al. 2003; Cummings 2006), and in the PVT for its role in attention processing (Allingham et al. 1998; Van der Werf et al. 2002; Heydendael et al. 2013). Again, there was a tendency for AD rats to have more basal Fos than CR in these regions. Although there was no main effect of diet, this higher basal neuronal activation meant stress only significantly activated ARC and PVT neurons in the CR rats (Table 2).

## Discussion

Chronic mild CR is known to be anxiolytic in behavioral tests of anxiety in rodents (Levay et al. 2007; Guccione et al. 2012; Willette et al. 2012) and to elevate circulating glucocorticoids under unstimulated conditions (Chacon et al. 2005; Levay et al. 2010; Tomiyama et al. 2010). In this study, we show that these effects of CR do not translate to substantially altered HPA axis sensitivity to psychological stress.

### CR is mildly anxiolytic

As previously demonstrated (Genn et al. 2003; Inoue et al. 2004), we showed rats fed 75% of their usual voluntary food intake entered the anxiety‐provoking open arms of the elevated plus maze more frequently than those fed an AD diet, indicative of less anxiety in this behavioral test. They also spent less time grooming in the open field, again indicative of reduced anxiety (Kalueff and Tuohimaa 2005), although a reduction in vertical exploration in this test may reflect enhanced anxiety. In any case, the anxiolytic phenotype in the CR rats was very mild and was specific to only some aspects of the behavioral tests. The CR rats did not spend more time in the open arms of the elevated plus maze, they did not explore the center of the open field more, nor did they show any anxiolytic behavior in the light/dark box.

This mild protection from anxiety is somewhat consistent with our Fos data. Neuronal activation in the absence of a stress stimulus is consistent with arousal and attention and we did see trends towards fewer activated cells under basal conditions in most regions activated. This possibly indicates the apparently anxiolytic phenotype is linked to less arousal and attention to the task when the rat is not stressed. Our findings from these behavioral tests also broadly concur with those of Levay and colleagues (Levay et al. 2007) in that chronic CR is somewhat anxiolytic, although test‐specific performance differed between the two studies. While we show evidence of CR causing less anxiety in the elevated plus maze, and an anxiolytic effect only on grooming in the open field, in Levay's study mild (25%) and severe (50%) CR reduced anxiety in the open field but not the elevated plus maze (Levay et al. 2007). These differences may reflect strain differences (Hooded Wistars were used in the Levay study (Levay et al. 2007)) or subtle experimental differences. Nonetheless, the data are collectively consistent, showing a mild anxiolytic effect of chronic CR. These findings also highlight the importance of conducting more than one behavioral test to examine anxiety behavior.

As with the Levay study (Levay et al. 2007), our elevated plus maze results do not seem to reflect enhanced food seeking behavior as we did not see a general increase in locomotor or exploratory behavior in any test. However, this possibility remains to be tested directly. One potential limitation of the present study is that the CR rats were given their food daily at 2 h after lights on. This feeding regime was deliberately chosen so that the behavioral tests would not be conducted on a background of fasting to avoid a group‐specific food‐seeking element. However, this feeding regime also meant the rats were given their daily food allocation outside their preferred feeding time (night/dark). In this regard, we should note 75% restriction is a mild calorie restriction and the rats do not eat all of the food immediately or even during the light phase. In all cases, there was food remaining when cages were checked in late afternoon. It is, thus, unlikely the behavioral tests included a food‐deprivation‐induced food‐seeking component in the CR or that the CR regime induced a phase‐shift in corticosterone rhythms.

### CR effects on HPA axis responses to stress

Also consistent with previous findings (Armario et al. 1987; Heiderstadt et al. 2000; Chacon et al. 2005; Levay et al. 2010), chronic mild CR led, in our study, to elevated basal circulating corticosterone. High levels of circulating glucocorticoids are a known risk factor in psychopathologies relating to stress, such as anxiety and depression (de Kloet et al. 2005). This elevated corticosterone coupled with the idea that a CR diet is difficult to maintain and that most organisms choose, instead, to eat to satiety (Marinilli Pinto et al. 2008) led us to hypothesize chronic mild CR would increase the sensitivity of the HPA axis to stress and result in exacerbated stress responses. Unexpectedly, HPA axis responses to stress remained relatively unaffected by CR. Briefly, stress activates CRH neurons of the mpPVN stimulating them to release CRH into the median eminence where it stimulates ACTH release into circulation. ACTH then acts on the adrenal cortex to initiate glucocorticoid release. Glucocorticoids combat the effects of the stressor and also negatively feed back onto the brain, principally at the hippocampus and the hypothalamus, to dampen further HPA axis activation (Sapolsky et al. 2000). The PVN is also reciprocally connected with regions of the brain responsible for feelings of anxiety and depression, such as the amygdala. As such, excessive stimulation of the PVN can stimulate amygdala‐related anxiety processes and amygdala activation can enhance PVN responses to stress (Pego et al. 2010). In the mpPVN, the apex of the HPA axis, and in all other stress‐sensitive brain regions examined, neuronal activation after stress was similar between CR and AD rats. We did see a significant interaction between stress and diet in the mpPVN that was likely accounted for by slightly less neuronal activation under basal conditions in this region in CR than AD rats and slightly more neuronal activation after restraint in the CR. There were no basal or poststress differences between the groups with post hoc tests, but these findings are nonetheless interesting. Fos is a marker of neuronal activation that is expressed in response to a stimulus. It is likely the Fos expressed in the mpPVN of nonstressed rats was induced by either the change in the light phase or by the handling/blood sampling procedures. In this regard, one could conclude that CR rats respond very mildly to a non‐stress stimulus, but an additional stressful stimulus is capable of inducing more of an mpPVN response. With this caveat in mind, however, any differences between the groups in neuronal activation after stress were not sufficient to be distinguished with post hoc analyses and were also not sufficient to effect downstream changes in the glucocorticoid response, making it unlikely CR is substantially stress‐provoking. Interestingly, we have previously seen the gastrointestinal peptide, ghrelin, elevated during fasting, is able to protect against stress (Spencer et al. 2012). The possibility therefore remains for such feeding‐related peptide hormones to counteract potential stress‐promoting effects of elevated glucocorticoids.

The present study is, to our knowledge, the first to directly examine HPA axis responses to stress after CR. However, previous groups have seen chronic mild CR leads to reduced basal CRH mRNA expression in the hypothalamus (Brady et al. 1990; Lindblom et al. 2005; Levay et al. 2010). It seems this change is insufficient to translate to a reduced HPA axis response to stress. Although we did not measure ACTH in this study, previous findings show ACTH is not altered under basal conditions in CR (Armario et al. 1987; Levay et al. 2010) and our findings suggest its responses to stress are also unlikely to be changed since glucocorticoid and PVN Fos levels were not different.

### Other models of calorie restriction

Levay and colleagues have shown the anxiolytic effects of 25% CR (as also used here) are very similar to those seen with a more severe 50% CR (Levay et al. 2007). Effects on basal glucocorticoids are statistically dose‐dependent, but the absolute differences in circulating corticosterone between 25% CR and 50% CR are small (Levay et al. 2010). The implication of these data is that a 50% CR would also not have substantial effects on the HPA axis response to stress. However, it is important to note that stress and HPA axis responsiveness are closely linked with adiposity in many models of weight loss and gain. Excess adiposity is associated with anxiety and depression in animal models of obesity, and in obese humans, and this is also usually reflected in exacerbated HPA axis responses to psychological stress (Doyle et al. 2007; Scott et al. 2008; Benson et al. 2009; Spencer and Tilbrook 2009; Abiles et al. 2010). Conversely, weight loss in obese populations can ameliorate HPA axis stress responses (Yanovski et al. 1997). We have also seen CR in early life, as in rat pups suckled in large litters, leads to a long‐term attenuation of HPA axis responses to stress (Bulfin et al. 2011; Clarke et al. 2013). On the other hand, severe CR, such as with an involuntary starvation diet or anorexia nervosa, exacerbates stress responses (Fichter and Pirke 1986; Connan et al. 2007). From these data and the current study, it appears there is a threshold or set point associated with the HPA axis response to acute stress. This set point can be shifted in cases of excess adiposity (obesity) and is restored to normal by dieting (CR) in the obese but is not further altered by CR in initially normal‐weight individuals. Severe CR (starvation, anorexia), however, involves additional metabolic and psychological factors that will activate the HPA axis and exacerbate its responses to additional stress.

## Summary and Conclusions

This study is the first to directly investigate the effects of a chronic mild CR on HPA axis responses to stress. We find CR rats are somewhat less anxious than their AD counterparts but that their HPA axis responses to stress remain relatively unaffected. These data support those suggesting a generally beneficial effect of mild CR (McCay et al. 1989; Fontana et al. 2010; Weiss and Fontana 2011; Pallavi et al. 2012; Jackness et al. 2013), in that dieting subjects may be slightly less anxious and are likely to retain their capacity to mount an appropriate stress response.

## Conflict of Interest

None declared.
